# Non-clinical safety considerations on genome editing using the CRISPR/Cas system

**DOI:** 10.1016/j.gendis.2025.101785

**Published:** 2025-07-28

**Authors:** Parto Toofan, Mark Singh, Andrew Brooks, Keith McLuckie

**Affiliations:** Cell and Gene Therapy Catapult, 12th Floor Tower Wing, Guy's Hospital, Great Maze Pond, London, SE1 9RT, United Kingdom

**Keywords:** Cellular and gene therapy products, CRISPR/Cas, *Ex vivo*, Gene editing, *In vivo*, Non-clinical safety

## Abstract

Recent advances in gene editing using the CRISPR/Cas system have revolutionized genome editing, opening new horizons for human cellular and gene therapy products. Genome editing technologies are rapidly being adopted in clinical trials. However, critical non-clinical safety considerations are required to address challenges in translating research to the clinic. Here, we review current *ex vivo* and *in vivo* genome editing approaches using the CRISPR/Cas system and discuss the practical use of these methods in pre-clinical studies and in the clinic. We also discuss known limitations of genome editing in humans and the mitigation of risk factors associated with it from a non-clinical safety perspective. This review aims to aid researchers in acquiring a perspective that is essential for the safe translation of genome editing to the clinic.

## Introduction

Gene editing has become more efficient and precise since the first targeted genetic engineering approaches in yeast and mice in the 1970s and early 1980s^1^, ^2^, ^3^ and in human cells in the late 1980s.^4^ Considering the low precision of traditional gene targeting, recovery of the desired products requires powerful selection and thorough characterization.^5^ Current genome editing technologies make directed genetic manipulations possible in almost all types of cells and organisms.^6^ The study of DNA damage and repair has aided in the development of high-efficiency genome editing through targeted DNA double-strand breaks (DSBs), which stimulate the generation of recombination between homologous sequences during meiosis.^7^ Genome editing is now being utilized in several clinical trials, with an increase in the development of clustered regularly interspaced short palindromic repeats (CRISPR)/CRISPR-associated protein (Cas)-modified cell and gene therapies globally.

The development of an advanced therapy from the pre-clinical stage through first-in-human clinical trials is a complex process and requires the development of a robust non-clinical strategy. Non-clinical programs include preclinical proof-of-concept, mechanism-of-action, efficacy, and safety/toxicology studies, and beyond first-in-human to the marketing authorization stage. To achieve a clinically relevant investigational cellular and gene therapy product (CGT), critical non-clinical safety considerations need to be taken into account to explore the risks associated with the CRISPR/Cas gene editing tool. “*The aim of non-clinical study programs during the development of investigational CGTs is to provide sufficient information for a proper benefit-risk assessment for first-in-human (FIH) use*” (EMEA/CHMP/SWP/28367/07 Rev. 1).^8^ “*The nature and extent of non-clinical development depends on the specifics of the CGTs, the availability of relevant animal models, the clinical indication, targeted clinical population, the intended route of administration (ROA), and the treatment regimen*” (EMEA/CHMP/SWP/28367/07 Rev. 1).^8^ Non-clinical development can be designed using a risk-based approach, and “*the core battery of Safety Pharmacology studies can be conducted as stand-alone studies or included in the toxicity studies*” (CPMP/ICH/539/00).^9^ Depending on the CGT, “*suitable control groups should be considered based on the knowledge on cargo, and components of the CGT*” (CPMP/ICH/539/00).^9^ It is recommended to follow the guidance of the US Food and Drug Administration (FDA), such as “*Human Gene Therapy Products Incorporating Human Genome Editing; Draft Guidance for Industry*” (FDA-2021-D-0398),^10^ “*Human gene therapy for rare diseases; guidance for industry*” (FDA-2018-D-2258),^11^ “*Considerations for the design of early-phase clinical trials of cellular and gene therapy products*” (FDA-2013-D-0576),^12^ and “*Guidance for industry: preclinical assessment of investigational cellular and gene therapy products*” (FDA-2012-D-1038)^13^ when developing a CGT in the US. According to the European Medicines Agency (EMA) guidelines, “*pivotal non-clinical safety studies should be carried out in conformity with the principles of good laboratory practice (GLP)*”. In the non-clinical development of an investigational CGT, “*Guideline on quality, non-clinical and clinical aspects of medicinal products containing genetically modified cells*” (EMEA/CHMP/GTWP/125459/2006),^14^ “*Guideline on the quality, non-clinical and clinical aspects of gene therapy medicinal products*” (EMA/CAT/80183/2014),^15^ “*Note for guidance on the quality, preclinical and clinical aspects of gene transfer medicinal products*” (CPMP/BWP/3088/99)^16^ and, where applicable, ICH E11(R1) “*Guideline on clinical investigation of medicinal products in the pediatric population*” (EMA/CPMP/ICH/2711/1999)^17^ should be taken into consideration.

Here, we evaluate the use of CRISPR/Cas technology and its translatability to the clinic from a non-clinical perspective. We provide a list of ongoing and completed clinical trials utilizing this technology and highlight important safety considerations when developing CRISPR/Cas CGT products.

## Evolution of gene editing

Endonucleases, enzymes that can cleave DNA, have been essential tools for genetic manipulation and have revolutionized biological research since the early 1970s.^1^, ^2^, ^3^, ^4^ Advances in the understanding of DNA repair mechanisms have led to increased interest in adopting these mechanisms in the clinic via the use of engineered nucleases. To date, various approaches have been investigated to explore effective and safe systems for sequence-specific gene editing.

The utilization of restriction enzymes remains the gold standard for gene editing research, enabling the introduction of foreign DNA sequences. By cleaving at a specific site in the genome, restriction enzymes create a space where the desired new sequence can be introduced. Restriction enzymes, however, have critical drawbacks, such as limited sequence-specific availability. The recognition sequence that prompts them to cleave is usually between four and eight bases and can often arise several times in a genome, reducing their specificity. Over the years, improvements have been made in the accuracy of restriction enzymes and modifications, allowing them to recognize unique sequences in the genome. Meganucleases, a family of naturally occurring rare-cutting endonucleases, are examples with such unique sequences that utilize longer DNA recognition sequences.^18^

**Figure 1:**
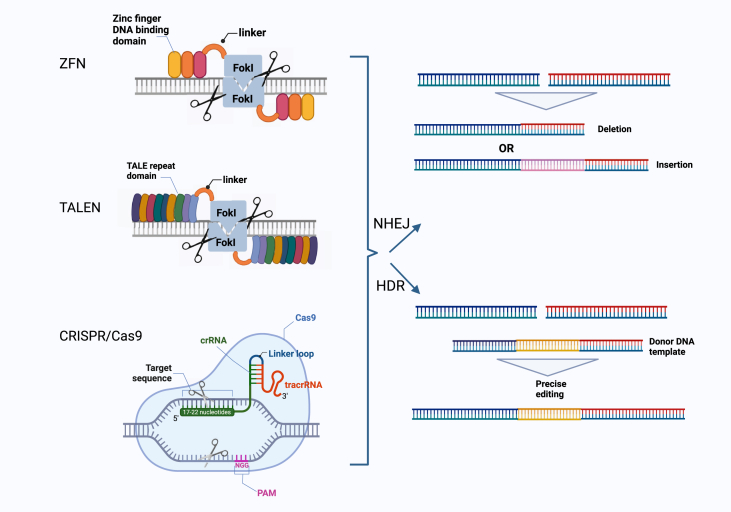
DSBs and DSB repair mechanisms using common genome editing platforms (ZFN, TALEN, and CRISPR/Cas9) (created with BioRender.com). DSB, DNA double-strand break; TALEN, transcription activator-like effector nuclease; ZFN, zinc finger nuclease; CRISPR, clustered regularly interspaced short palindromic repeats; Cas, CRISPR-associated protein.

Transcription activator-like effector nucleases (TALENs) and zinc finger nucleases (ZFNs) were the first programmable gene editing tools. TALENs and ZFNs consist of a non-specific flavobacterium okeanokoites I (FokI) nuclease domain and a tailor-made DNA recognition domain, which binds to the target site, allowing dimerization of the FokI endonuclease. As these methods involve engineering proteins to target the desired DNA region, they are both time- and labor-intensive^19^^,^^20^ (Fig. 1).

The clustered regularly interspaced short palindromic repeats (CRISPR)/associated protein (CRISPR/Cas) system is a robust and versatile platform that has undergone rapid adoption and development in recent years for gene correction, transcriptional regulation through gene activation and gene silencing, disease modeling, and nucleic acid labeling/imaging. The CRISPR/Cas system serves as an adaptive immune system in archaea and bacteria for protection from viruses. The CRISPR/Cas system has two essential components, guide RNA (gRNA) and the Cas protein, which enable effective genome editing in eukaryotic cells. Compared with TALENs and ZFNs, the CRISPR/Cas system is less labor-intensive with respect to protein design. SpCas9, derived from *Streptococcus pyogenes*, is the most used Cas protein due to its simplicity and high target specificity.^21^

## CRISPR/Cas9-mediated genome editing: An overview

**Figure 2:**
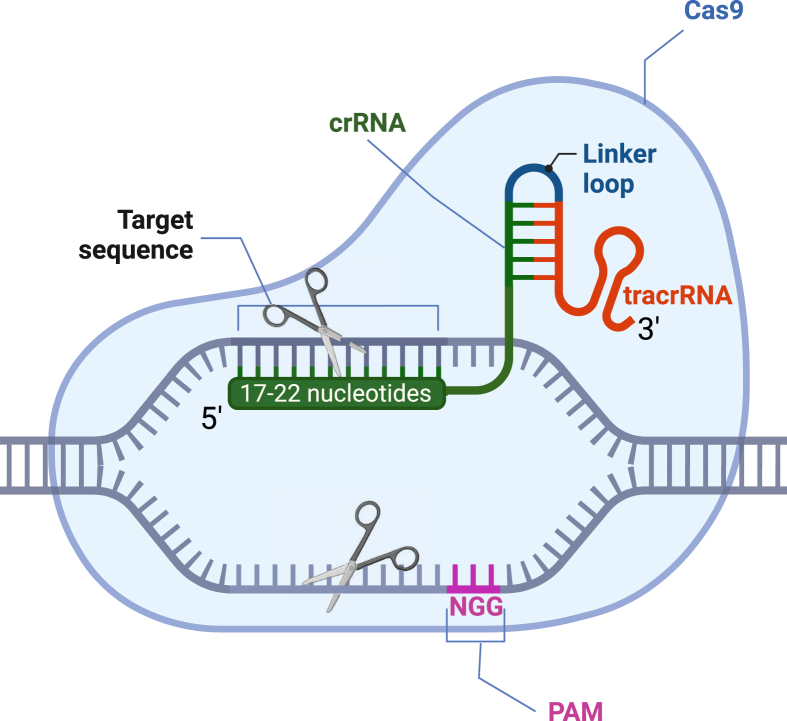
sgRNA components (created with BioRender.com).

CRISPR/Cas9 relies on two components for gene editing, a custom-designed small guide ribonucleic acid (sgRNA) that recognizes the DNA sequence of interest and “guides” the Cas9 protein to this locus to generate site-specific DSBs and a Cas endonuclease. sgRNAs can be generated *in vitro* or *in vivo* by fusing a crispr RNA (crRNA) and a tracer RNA (tracrRNA). A crRNA includes a complementary sequence to tracer RNA (tracrRNA), which serves as a binding scaffold for the Cas nuclease. By re-designing the crRNA component of the sgRNA, one can adapt this system to different genomic target sites. In addition, the CRISPR/Cas9 system is insensitive to the epigenetic state of the target site^22^ and allows cleavage at multiple sites by co-delivering diverse sgRNAs.^23^ The Cas9 protein contains two endonuclease active site domains, RuvC and HNH. The HNH nuclease initiates cleavage by cutting the DNA strand that is complementary to the crRNA, while the RuvC domain cleaves the opposite strand. A scaffold sequence on a sgRNA enables its anchoring to Cas9 and the sequence of interest. The sgRNA comprises a 17–20 bp sequence that is complementary to the target locus upstream of the protospacer adjacent motif (PAM) on the non-targeted DNA strand. The custom-designed sgRNA binds to and “guides” the Cas9 protein to the targeted locus, leading to site-specific DSBs^24^^,^^25^ (Fig. 2).

The canonical CRISPR/Cas9 editing method relies on two endogenous DNA repair mechanisms: error-prone non-homologous end joining (NHEJ) and more precise homology-directed repair (HDR). The former DNA repair mechanism occurs more frequently in most cell types and involves the generation of insertions/deletions (indels), creating premature stop codons and/or non-functional polypeptides, resulting from frameshift mutations. This pathway is useful in clinical applications where gene disruption provides a therapeutic opportunity. The latter repair mechanism uses a donor homology repair template for knocking in gene segments (Fig. 1).^26^^,^^27^ As DSBs can lead to large deletions, chromosomal translocations, retrotransposon insertions, and p53 activation, both outcomes may lead to undesired consequences and potentially enrich oncogenic cells.^28^, ^29^, ^30^, ^31^, ^32^, ^33^

## Advances in CRISPR/Cas gene editing

The CRISPR/Cas9 system has been used in various applications, including the correction of a single gene in monogenic disorders and oncogenic mutations (reviewed in Zhang et al, 2021).^34^ Recent advances in genetic engineering have enabled scientists to extend the applications of the CRISPR/Cas9 system by modifying the two nuclease domains. Designer Cas proteins have been developed by modifying the structure of wild-type Cas9 to generate nuclease-inactivated Cas9 (dead Cas9-dCas9) and “nickase” Cas9 (nCas9), which generate single-strand cuts with lower levels of off-target events (reviewed by Knott and Doudna).^35^ dCas9 can be utilized to control transcriptional/epigenetic regulation, such as gene silencing and gene activation.^36^ Furthermore, the high-fidelity enzyme SpCas9-HF1 reduces editing at off-target sites.^37^^,^^38^ Cas-CLOVER is another good example of a high-fidelity site-specific nuclease with low off-target activity utilized for the safer and more robust generation of stem cell-like memory T-enriched allogeneic chimeric antigen receptor (CAR)-T cells.^39^

Base editing is another advancement in the application of CRISPR/Cas technology.^40^^,^^41^ dCas9 or nCas9 can be fused to DNA base-modifying enzymes to facilitate the editing of single bases. This is achieved through the conversion of a single base into another without generating DSBs or using a donor template. By fusing CRISPR/Cas9 to a cytidine deaminase enzyme, cytidine is converted to uridine, resulting in a C–T transition.^40^ Another good example of base editing without generating DSBs is the use of adenine base editors, which mediate the conversion of A-T to G-C in genomic DNA with high efficiency and a low indel rate.^41^ AYBE, an adenine transversion base editor for A-to-C and A-to-T transversion editing in mammalian cells, is generated by fusing an adenine base editor with the hypoxanthine excision protein N-methylpurine DNA glycosylase (MPG).^42^ ACBE is another example of base editors for simultaneous substitutions of C-to-T and A-to-G in mammalian systems and is generated by fusing a heterodimer of TadA (ecTadAWT/∗) and an activation-induced cytidine deaminase to the N- and C-termini of nCas9, respectively.^43^

Prime editing is another versatile tool for precise genome manipulation, without the direct requirement for DSBs (reviewed by Chen and Liu).^44^ Prime editing requires a prime editor protein (usually a fusion of nCas9), a reverse transcriptase, and a prime editing gRNA (pegRNA), which specifies the target site for the edit and contains a programmable RNA template for the desired genetic change. Prime editing enables DNA sequence substitution, small insertions, and small deletions without the direct need to generate DSBs.^44^ In a concise review, Chen and Liu described the capabilities and limitations of prime editing and discussed opportunities for further enhancement of this technique.^44^

With knowledge of the cell cycle and DNA repair mechanism components, it is possible to direct the cell machinery toward the desired outcome. By suppressing the NHEJ pathway via the use of chemical inhibitors of the key enzymes in this pathway, it is possible to increase HDR efficiency. Exploiting cell cycle phase control can also be used as a means to favor templated repair to increase HDR edits while preventing undesired NHEJ events. During the resolution of DSBs, the two main DNA repair mechanisms, HDR and NHEJ, compete, and depending on the cell cycle phase and availability of the components, including a repair strand, one will be favored over the other. In eukaryotes, NHEJ predominantly occurs in the G1 phase of the cell cycle (reviewed by Chang et al).^45^ In human cells, HDR is predominant during the S and G2 phases of the cell cycle.^46^^,^^47^ Furthermore, to enhance HDR in human cells, Cas9 protein fusions can be generated by biasing the DNA repair pathway choice at the site of the DSB.^48^, ^49^, ^50^

Controlling the transcription level of a single gene or multiple genes by fusing transcriptional activators and repressors with Cas enzymes has made the CRISPR/Cas technique more versatile than ever.^19^^,^^51^ Cas9 and Cas12a^52^ are the Cas proteins frequently used for regulating gene editing and transcriptional regulation.^52^ Like other Cas proteins, the mechanism of action of these Cas variants is via Watson‒Crick base pairing between the target sequence and gRNA. Additionally, the gRNA for Cas12a is referred to as crRNA.^21^^,^^52^ The efficiency of editing can be further enhanced by fusing dCas9 and dCas12a to effector domains to enable CRISPR-mediated inhibition (CRISPRi) and activation (CRISPRa).^53^^,^^54^

Simultaneous targeting of multiple genomic sites has been possible for both CRISPR-mediated gene editing and transcriptional regulation. By introducing multiple gRNAs targeting different sites in the genome, it is possible to perform multiplex genome editing via CRISPR technology.^23^ In addition to enhancing editing efficiency or regulating transcription at one site, Cas enzymes can be guided by multiple crRNAs to target a single genetic locus.^55^ In a concise review, McCarty et al compared methods of multiplex genome editing and transcriptional regulation using CRISPR/Cas techniques, discussed multiplexed CRISPR technologies, and described methods used for gRNA arrays *in vivo*.^51^

## Application of CRISPR/Cas in the clinic

**Figure 3:**
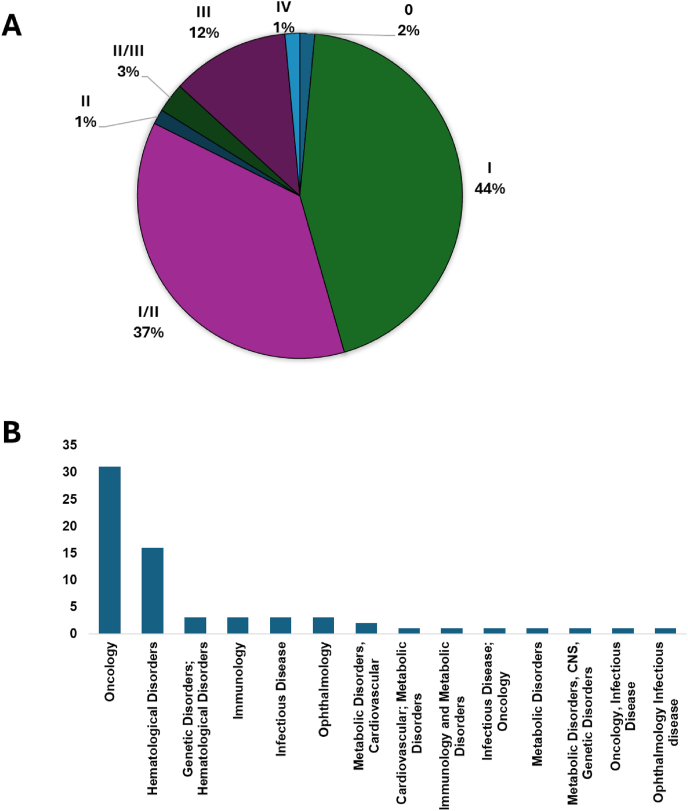
Clinical trials utilizing CRISPR/Cas9 **(A)** Number of clinical trials between 2015 and 2023 presented by phase in percentage. **(B)** Number of clinical trials presented by therapy area. Data were extracted and combined from ClinicalTrials.gov and GlobalData on 21st August 2024. CRISPR, clustered regularly interspaced short palindromic repeats; Cas9, CRISPR-associated protein 9.

The GlobalData and ClinicalTrials databases were used to identify clinical trials involving CRISPR/Cas technology initiated from 2015 to 2024. A data search was performed on 12 June 2025 on all registered global clinical trials containing at least one of the following terms: CRISPR, Cas9, CRISPR/Cas, CRISPR-CAS genome-edited T cells, CRISPR‒Cas genome-edited natural killer (NK) cells, and CRISPR/Cas others. The combined results from both databases are presented in Table S1. A total of 68 ongoing, completed, terminated, and withdrawn clinical trials utilizing CRISPR/Cas9 technology were identified. Three studies have been registered using Cas12a (NCT04853576, NCT05444894, and a recent base editing trial by Beam Therapeutics), and one study has been registered using Cas13. Since the development of CRISPR/Cas9, there has been a significant improvement in the efficiency and safety of this technique. While the number of clinical trials in this field has increased in recent years, most of these trials are in phases I and I/II, where safety and treatment side effects are evaluated (Fig. 3A). Current registered trials are underway in 9 therapy areas: cardiovascular disorders, developmental disorders, ophthalmologic disorders, immune disorders, metabolic disorders, oncology, infectious diseases, hematological disorders, and genetic disorders. Most CRISPR/Cas9 clinical trials are in the oncology therapy area (Fig. 3B), in which T cells are harnessed for adoptive cell transfer (Table S1). Recent successes in the field of gene editing using CRISPR include the US FDA-granted approval of CASGEVY® to Vertex Pharmaceuticals, Inc., and the approval of LYFGENIA to Bluebird Bio, Inc. Both gene therapies received Priority Review, Orphan Drug, Fast Track, and Regenerative Medicine Advanced Therapy designations. CASGEVY®, a non-viral *ex vivo* cell-based gene therapy delivered via the intravenous route, is approved for the treatment of sickle cell disease in patients aged 12 years or older with recurrent vaso-occlusive crises and β-thalassemia who need regular blood transfusions. LYFGENIA is an *ex vivo* cell-based gene therapy utilizing a lentiviral vector approach that is administered via intravenous infusion and is approved for the treatment of patients aged 12 years or older with sickle cell disease and a history of vaso-occlusive events.

Currently, CRISPR/Cas gene editing is utilized to genetically manipulate different types of adoptive cell transfer, including general T cells, tumor-infiltrating lymphocytes, T-cell receptors (TCRs), and CAR-T cells. This gene editing technique is generally applied to adoptive cell transfer to increase survival after transfer, increase efficacy, prevent self-targeting, or develop universal T cells.

The earliest clinical trial utilizing CRISPR/Cas9 gene editing (NCT02793856) focused on knocking out the programmed cell death protein 1 (PD-1) gene in peripheral blood T cells using Cas9. PD-1 is expressed on the surface of activated T cells and negatively regulates T-cell activation upon interaction with its ligand, PD-L1. As high expression of PD-1 on the T-cell surface accelerates T-cell tolerance and exhaustion, it leads to reduced efficacy of T cells against tumors. The primary safety concern in this trial was the use of non-specific T cells, which might lead to overactivation of the patient's immune system^56^; however, no major adverse events were reported in this study.

The use of tumor-infiltrating lymphocytes collected directly from patient tumor resections has been reported to result in long-term robust patient response in patients with melanoma^57^ and is currently being used in four clinical trials (NCT04426669, NCT03538613, NCT04089891, and NCT05566223). These autologous tumor-infiltrating lymphocytes with enhanced anti-tumor efficiency are generated by knocking out the gene cytokine-inducible SH2-containing protein (CISH) via CRISPR/Cas9 in patient-derived tumor-infiltrating lymphocytes once they are collected from tumors. CISH is a member of the suppressor of cytokine signaling (SOCS) family and has been shown to reduce tumor-infiltrating lymphocytes' activity against cancer through blocking their avidity.^58^

The development of TCR therapy has enabled patients' T cells to express TCRs that recognize one or multiple combinations of specific cancer peptides, effectively eliminating tumor cells.^59^ A potential limitation to TCR therapy is the mispairing of the TCR α and β chains with the engineered TCR α and β chains, which creates heterologous pairs. These heterologous pairs might reduce treatment efficiency and cause toxicity due to off-target off-tumor binding.^60^ NY-ESO-1 (NCT03399448) is one of the most promising autologous therapies in the oncology area for treating melanoma, synovial cell carcinoma, and myeloma.^61^^,^^62^ This gene editing trial focused on engineered TCRs generated via CRISPR/Cas9 to knock out endogenous TCR α/β chains and PD-1 to reduce the potential for generating heterologous pairing and enhance T-cell efficiency, respectively.^63^

The use of CAR-T-cell therapy has been promising in both hematological disorders and solid tumors.^64^^,^^65^ However, there are several limitations when CAR-T-cell therapies are used, including insufficient quantity and poor quality of autologous T cells, CAR-T-cell exhaustion, tumor suppressive microenvironments, and potential self-killing and uncontrollable proliferation. There are currently thirteen ongoing/completed early-stage clinical trials using CRISPR/Cas9-modified universal CAR-T cells (Table S1). CRISPR/Cas9 genomic editing technology, which is flexible, simple, highly efficient, and multiplexed, holds promising avenues for the generation of next-generation CAR-T-cell products. This includes the generation of “off-the-shelf” universal CAR-T cells by disrupting human leukocyte antigen (HLA) or endogenous TCRs, the generation of more potent CAR-T cells via the ablation of multiple inhibitory modulators, and the generation of more controllable next-generation CAR-T cells via the addition of inducible safe switches or suicide genes.^66^

In an attempt to produce universal CAR-T cells, CRISPR/Cas9 gene editing was used to deplete the endogenous TCR, both the α and β chains, to avoid graft versus host disease. In this study, Ren et al developed an efficient method for multiplex genome editing by incorporating multiple gRNA cassettes into a single CAR lentiviral vector to generate DKO UCART19 cells.^67^ The results demonstrated high anti-leukemic activity and no induction of graft versus host disease in the NSG mouse model when this universal T-cell was combined with the depletion of other genes via CRISPR/Cas9.^67^ In a recent phase I clinical trial (NCT04557436), the safety of CRISPR/Cas9 gene-edited allogeneic CD19 CAR-T cells is being evaluated in pediatric subjects with relapsed or refractory B acute lymphoblastic leukemia. In this study, T cells are transduced to express an anti-CD19 CAR (CAR19) via a lentiviral vector that also incorporates CRISPR/Cas9 genome editing of CD52 and TRAC to increase resistance to depletion and enhance potency, respectively. The transient expression of Cas9 in this system limits the risk of off-target effects due to the limited expression time. The efficiency of CAR-T cells can be further improved by 80 % through the simultaneous targeting of endogenous TCRs and B2Ms.^67^ Knocking out B2M has been adopted in a clinical trial in 2017 (NCT03166878), where universal CD19 CAR-T cells were generated by further modification using CRISPR/Cas9 to deplete B2M expression.

Owing to their cell-mediated cytotoxic properties against hematological malignancies and anti-tumor potential, NK cells, as potential next-generation adoptive immunotherapy tools, have demonstrated better safety profiles than CAR-T cells in preliminary clinical trials.^68^ All these findings contribute to the increasing number of clinical trials assessing engineered NK cell products. Recent strategies have focused on increasing NK quantity and improving their ability to overcome cancer resistance in the immunosuppressive tumor microenvironment. Good examples of such strategies include boosting NK propagation and killing capacity using cytokines and synthetic compounds, targeting immune function checkpoints, adding CARs to NK cells to provide cancer specificity, and genetic ablation of inhibitory molecules.^69^^,^^70^ The main obstacle in the clinical translation of NK cells has been the difficulty in obtaining sufficient quantities for treatment and the challenges in enhancing their function and persistence *in vivo*.^71^^,^^72^ Induced pluripotent stem cells (iPSCs), which have limited self-renewal capacity and high differentiation potential, have enabled scientists to overcome cell source obstacles. Furthermore, CRISPR/Cas9 technology has been shown to be a successful approach for editing both iPSCs and NK cell receptors. Recent advances in the use of iPSCs and CRISPR/Cas9 editing tools have supported the creation of large quantities of gene-edited NK cells with improved functionality.^73^, ^74^, ^75^, ^76^, ^77^, ^78^, ^79^ The US FDA cleared the Fate Therapeutic's Investigational New Drug (IND) application for FT538 (NCT04614636) in 2020, the first CRISPR-edited iPSC-derived cell therapy. This iPSC-derived off-the-shelf NK cell cancer immunotherapy is engineered to have novel high-affinity, non-cleavable CD16 (hnCD16) Fc receptor; an IL-15/IL-15 receptor fusion (IL-15RF); and knockdown of the CD38 gene via CRISPR/Cas9 to eliminate daratumumab-induced CD38^+^ immune regulatory cell depletion, which in turn increases NK cell effector activity. This therapy is planned to be used as a monotherapy in acute myeloid leukemia and in combination with daratumumab, a CD38-directed monoclonal antibody therapy for the treatment of multiple myeloma.

## Non-clinical risk mitigations on CRISPR/Cas-generated CGT products

Advances in the CRISPR/Cas system have provided hope for the correction of a wide range of human disorders. However, for safer clinical translation of this technique, an appropriate developmental plan must be carried out. Table 1 summarizes the necessary considerations when planning non-clinical studies for the development of a CRISPR/Cas-generated CGT. Considering the constraints associated with *in vivo* models, regulatory and health authorities endorse the development of novel approach methodologies, which are innovative chemistry-based methods, and *in silico*, *in vitro*, and *ex vivo* approaches intended for the safety/toxicology evaluation of novel therapies, with the goal of replacing traditional *in vivo* testing requirements.^80^

**Table 1:** Non-clinical risk mitigations for CRISPR/Cas cellular and gene therapy products.

Item	Risks/challenges	Mitigation
Animal model selection	Animal model, age, and gender	Intended patient population and the type of drug product determines the animal model, age, sex, and necessity of developmental and reproductive toxicity studies. The goals and limitations of *in vivo* studies should be defined. If the transgene product does not demonstrate equivalent pharmacology, then the use of the animal equivalent product has regulatory precedent.
Toxicity	Possible autoinflammatory and autoimmunity, graft versus host disease, cytokine release syndrome/tumor lysis syndrome, on-target off-tumor (non-target tissue), and off-target off-tumor effects. The toxicity of the drug product is influenced by route of administration, dosing regimen, and distribution. Other aspects include chemistry, manufacturing, controls, and any associated risks.	Any undesired effects of the product, and the doses at which they occur, in general toxicity assessment and histopathology examination, should be identified. The use of the same animal model in both the toxicity investigations and efficacy/pharmacodynamics studies may allow correlation of the biodistribution of the candidate cell product with observed toxicity signals.^128^ Pivotal toxicity (and biodistribution) studies should be performed under OECD principles of good laboratory practice (GLP), or otherwise justified and agreed with regulatory authorities.^128^
Immunogenicity	Induction or modulation of the patient's immune response by bacteria-derived products (Cas/RNA/DNA) or novel lipid nanoparticles leading to potential product clearance, decrease of efficacy, and eventually treatment failure. Immunogenicity is affected by route of administration and biodistribution.	There is a need for extensive knowledge on the mechanism of action, product characteristics, production methods, and non-clinical assessment limitations.^129^*In vitro* assessment, such as cytokine and chemokine release assay and blood serum cytotoxicity test, can be employed. *In silico* approaches can help identify potential immunogenic domains.
Biodistribution, persistence, and clearance	Differential target expression and long-term persistence of allogeneic/autologous product. Biodistribution, persistence, and clearance are influenced by route of administration and dosing.	The distribution, persistence, and clearance of cellular and gene therapy products *in vivo* in all organs, in both target and non-target tissues, should be investigated. Use of immunodeficient animal models allows the long-term biodistribution, persistence, and clearance study of human cells and any associated toxicities.
Tumorigenicity	Off-target mutagenesis, genetic instability, and where applicable undifferentiated iPSC. Risk of tumorigenicity is affected by route of administration, biodistribution, and dosing strategy.	*In silico* tools for gRNA design can be used for off-target assessment. The selectivity of editing can be confirmed using a quantitative method such as digital droplet PCR and next-generation sequencing. A tumorigenicity late endpoint should be included in the toxicity/biodistribution studies or performed as a separate study. Karyology and assessment of the copy number variations need to be included when developing induced pluripotent stem cell-derived products. Full characterization of the final product and functional assays will capture the risk of unwanted cell populations/clones.

Note: CRISPR, clustered regularly interspaced short palindromic repeats; Cas, CRISPR-associated protein.

The delivery route of a cell or gene therapy has a great impact on its safety and therapeutic efficiency. Whether *in vivo* or *ex vivo*, delivering CRISPR/Cas components to target organs or cells remains a challenge. Utilizing viral vectors for *in vivo* gene therapy is clinically challenging. Common viral vectors used for the delivery of the CRISPR/Cas9 system in an *ex vivo* or *in vivo* setting include adenovirus, adeno-associated virus (AAV), and lentivirus (Table 2). Considering their low immunogenicity, stable transgene expression, serotype-related targeting, and non-integrative characteristics, AAVs have been the preferred choice among viral vectors. Immune-privileged organs, such as the eye, have great potential for *in vivo* gene therapies using AAVs as the vector. In the first *in vivo* clinical trial using CRISPR/Cas9 (EDIT-101, NCT03872479),^81^^,^^82^ the AAV5 vector was used to deliver CRISPR/Cas9 components to correct CEP290 mutations in photoreceptor cells. In this study, the AAV5 vector was delivered via the subretinal route to the eye to treat Leber congenital amaurosis and retinal degeneration (Table S1). In this system, to circumvent off-target editing, a promoter-control design is employed to enhance cell selectivity. Although no serious adverse events related to the treatment or procedure and no dose-limiting toxic effects have been recorded,^82^ EDIT-101 is currently paused, as efficacy is only observed in patients with Leber congenital amaurosis homozygous for IVS26 mutation, which represents a very small population of the disease.

**Table 2:** Comparison of viral approaches in the application of CRISPR/Cas9.

Vector	Advantages	Disadvantages	Maximum cargo size
**Adeno-associated virus**^**130**^	• Low immunogenicity	• Low packaging capacity	4.5 kb
	• Serotype-related targeting		
	• Stable transgene expression		
	• Episomal; expression is diluted proportionally to the cell division.		
**Adenovirus**^**131**^	• Large packaging capacity	• High immunogenicity	>8 kb
	• Non-integrating		
	• Episomal; expression is diluted proportionally to the cell division.		
**Lentivirus**^**120**^	• Large packaging capacity	• Integrating into the genome with long-lasting expression of Cas9	8 kb
	• Application to dividing and non-dividing cells		

Note: CRISPR, clustered regularly interspaced short palindromic repeats; Cas9, CRISPR-associated protein 9.

The packaging limitations associated with AAV vectors (∼4.5 kb) render the simultaneous encapsulation of both the Cas9 sequence and sgRNA in a single AAV vector challenging. This limited capacity can be partially overcome using truncated SpCas9, SpCas9 fragments, or other Cas9 orthologs, such as *S. aureus* Cas9 (SaCas9).^37^ Furthermore, intein-mediated protein trans-splicing has been shown to increase the AAV transfer capacity in the retinas of mice, pigs, and human retina organoids.^83^ High doses of AAV may increase the risk of toxicity, immunogenicity, and other adverse events. AAVhu68-based gene therapy via high-dose intravenous injection for spinal muscular atrophy led to marked elevation in transaminase levels and acute liver toxicity in nonhuman primates.^84^ Similarly, two cisterna magna-delivered AAV9-based gene therapies for Hunter syndrome and Hurler syndrome resulted in minimal to moderate asymptomatic axonopathy at both low and high doses.^85^ A prophylactic course of corticosteroids was administered to patients to suppress the immune system and ameliorate hepatocellular toxicity.^86^ Studies have shown that up to 6 weeks of immunosuppression using corticosteroids is sufficient to control transaminitis.^87^ Although high-dose intravenously delivered AAV9 (2 × 10^14^ genome copies/kg) has been associated with severe toxicity in juvenile nonhuman primates and piglets,^84^ a 4-year nonhuman primate study in which a lower dose of AAV9 (2 × 10^12^ genome copies/kg) was administered intrathecally revealed no detectable toxicity and sustained exposure of the transgene over four years.^88^ The other consideration with AAV vectors is pre-existing immunity against AAV, which would lead to reduced efficacy by invalidation of transfection via antibody neutralization.^89^ An alternative option is the use of adenovirus, which is a non-integrating double-stranded DNA virus with the ability to transfect both dividing and non-dividing cells. *In vitro* validation of a CRISPR/Cas9-mediated gene correction strategy for cystic fibrosis has been performed in pig cells, which demonstrated the precise integration and persistent functional expression of the human cystic fibrosis transmembrane conductance regulator (CFTR) gene. This breakthrough, which utilizes helper-dependent adenoviral vectors for gene transfer, provides critical preclinical data supporting the feasibility of this approach for permanent CFTR correction and sets the stage for future *in vivo* studies in pig models, overcoming previous limitations in gene therapy delivery and sustained therapeutic expression.^90^ Adenovirus has a greater capacity compared with AAV (28–38 kb),^91^ which enables simultaneous delivery of the Cas9 sequence together with multiple sgRNAs in one vector for multiplex genome editing; however, adenovirus has a relatively high risk of immunotoxicity and immunogenicity.^91^

Lentivirus approaches result in stable expression, increased packaging capacity (∼8 kb), and excellent infection efficiency in both dividing and non-dividing cells. Nevertheless, this method has a higher rate of insertional mutagenesis and a greater chance of off-target mutagenesis. The recent emergence of the integration-defective lentivirus developed by generating point mutations in the integrase protein of the lentivirus may mitigate the potential risk of inherent insertional mutagenesis.^92^ Although integration-defective lentivirus shows lower transgene expression compared with its integrating counterpart, the expression levels of integration-defective lentivirus have been shown to be improved by 6–7-fold when the IS2 element is inserted.^93^

Owing to their applicability and transfection efficiency, viral vectors hold great potential for *in vivo* genome editing. The obstacles to address in clinical use include, but are not limited to, insufficient targeting in systemic administration, immunogenicity of the Cas protein and viral vector, packaging capacity limitations, and poor control of editing. These limitations have promoted the delivery of Cas9 mRNA and gRNA, plasmid DNA (pDNA), and Cas9 ribonucleoprotein (RNP) cargos via nonviral delivery systems (Table 3).

**Table 3:** Comparison of non-viral delivery cargo in the application of CRISPR/Cas9.

Carrier (cargo)	Advantages	Disadvantages
**Plasmid DNA**	• Simplicity	• Delayed onset
	• High stability	• Integration risk
**RNA system**	• Quick onset	• Rapid degradation
	• Non-integrating	
	• Low off-target effects	
**Ribonucleoprotein**	• Quick onset	• Instability
	• Non-integrating	• Structural complexity
	• Low off-target effects	

Note: Non-viral cargos can be delivered via physical or chemical methods. The selection of the cargo and mode of delivery is determined by the *in vitro*, *ex vivo*, and *in vivo* status of the treatment. CRISPR, clustered regularly interspaced short palindromic repeats; Cas9, CRISPR-associated protein 9.

Lipid nanoparticles (LNPs) have been used in a phase I clinical trial (GDC30025382) to deliver Cas9 nuclease and gRNAs to the *TTR* gene for the treatment of several cardiovascular and metabolic disorders (Table S1). This gene therapy product is administered via parenteral routes, including subcutaneous, intraperitoneal, intradermal, intravenous, and intramuscular routes, and eliminates the production and accumulation of the mutated TTR protein by cleaving the aberrant TTR gene. The non-clinical development of a CGT encapsulated in a novel LNP requires a full safety toxicology assessment of the LNP component prior to its first use in humans.^94^ Planned clinical trial NCT06155500 by Novartis AG, an *ex vivo* autologous cell therapy, and the ongoing clinical trial NCT06128629 by Intellia Therapeutics, Inc., an *in vivo* gene therapy, also take advantage of LNP delivery via the intravenous route. In a recent review, Hou et al discussed the use of LNPs in mRNA delivery, associated challenges and mitigations, and their translatability to the clinic.^95^ In a recent review, Palacios et al discussed the *in vivo* application of CRISPR/Cas9 genome editing and its opportunities and limitations.^96^

Recent advances in base editing have led to the success of two phase I completed (NCT05398029) and ongoing (NCT06164730) clinical trials by Verve Therapeutics, Inc., where *in vivo* CRISPR genome editing therapy comprises an adenine base editor encoded by an mRNA and an optimized gRNA packaged in an LNP delivery system targeting the liver proprotein.

### DNA damage toxicity, apoptosis, and tumorigenicity involving P53

Recent data have revealed p53-mediated cell toxicity and possible tumorigenicity associated with gene editing that requires DSBs.^97^ Unwanted outcomes associated with the activation of the p53 pathway include the induction of apoptosis rather than the intended gene editing or positive selection of p53-mutant cells, thereby having tumorigenic potential. Recent data show that p53-mediated toxicity is highly variable depending on the DNA sequence of interest and the epigenomic state in the region surrounding this locus.^97^ As also shown by others, DSBs at target sequences that are located in active, accessible chromatin lead to a stronger p53-mediated toxic response.^98^ Currently, there is no publicly available tool to mitigate this problem; however, a custom-designed sgRNA library may detect regions with high toxicity profiles for a specific CGT, and the activation of p53 pathways can be assessed *in vitro* through various molecular biology, genomic, or immuno-imaging techniques.^99^

### In silico assessment of on-target and off-target editing

The potential for off-target mutations and immunogenicity of the Cas9 protein are two major hurdles in the use of this system for *in vivo* human use. Computational methods can be applied to design sgRNAs with high efficiency (on-target scores) and specificity (off-target scores). The use of bioinformatic tools is essential in designing gRNAs in the context of multiplexing CRISPR. When multiple gRNAs are targeted to edit or regulate numerous genetic loci in tandem, it is important to investigate all possible target sites, as the genome in question is scanned to find a 17–20 bp region adjacent to a specific PAM site. In such scenarios, it is challenging to predict the on- and off-target scores of each gRNA *de novo*. The on-target score of a gRNA is defined as a measure of its ability to bind to its specific target site, and off-target scores, as the name suggests, predict the likelihood of the gRNA binding to undesired positions in the genome.^100^ Using Sniper-Cas9 and the “Sniper-screen” tool aids in obtaining a Cas9 variant with optimized specificity and retained on-target activity.^101^^,^^102^ In addition to the assessment of indel cutting efficiency, recent algorithms have improved gRNA efficiency by incorporating gRNA stability, Cas9 loading, chromatin, position, and sequence features of the genetic locus into the predictive model.^103^^,^^104^ Biophysical models for CRISPR/Cas9 activity have also been developed, leveraging statistical mechanics and kinetics to model each step of the editing process and factor in Cas9-mediated cleavage, RNP formation, and DNA supercoiling.^105^ Furthermore, new emerging deep learning models such as ABEdeepoff and CBEdeepoff are under development for the prediction of base editor off-target effects,^106^ and *in vitro* assays such as tagmentation of prime editor (TAPE) sequencing have been developed to predict genome-wide off-target effects of prime editors.^107^ There are also online databases, including WeReview: CRISPR Tools, where updated lists of bioinformatics tools are available for CRISPR/Cas experiments.

### CRISPR/Cas9-associated immunogenicity

The immunogenicity and immunotoxicity of an investigational CGT should be investigated and addressed in non-clinical studies before eliciting human exposure regimens. Both SaCas9 and SpCas9 are prevalent in human commensals that could be pathogenic. *Ex vivo* applications of CRISPR/Cas9 are unlikely to provoke an immune response, as Cas9 is an intracellular protein, and anti-Cas9 antibodies would be hypothetically negligible.^108^ Given the bacterial origin of Cas proteins and the potential immunogenicity of viral vectors, the overall potential for immunogenicity of CRISPR/Cas9 CGT should be perceived as a relevant risk to further development beyond first-in-human clinical trials. Strategies for mitigating any immunogenicity, such as attenuation of therapeutic effects or side effects for *in vivo* genome editing via CRISPR/Cas9, should be built into the development program.

**Figure 4:**
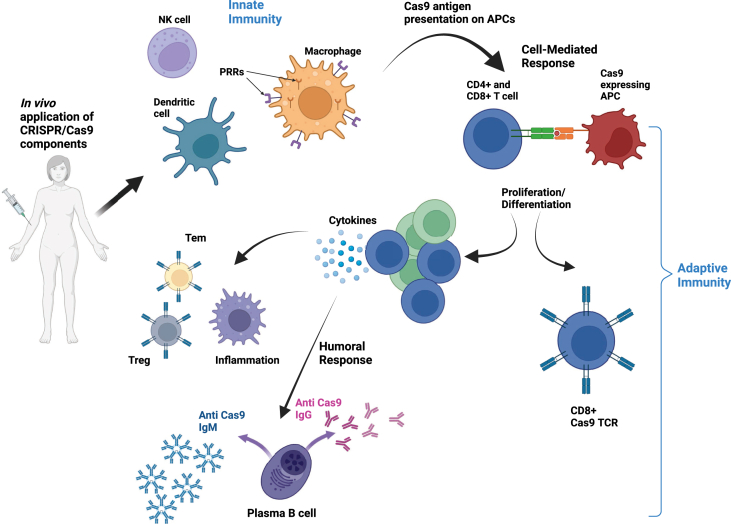
Activation of innate and adaptive immunity in response to Cas9 (created with BioRender.com). Cas9, CRISPR-associated protein 9.

*In vivo* delivery of CRISPR/Cas9 components can trigger both innate and adaptive immune responses (Fig. 4). Pattern recognition receptors (PRRs) recognize conserved features of foreign proteins and nucleic acids, which ultimately leads to the activation of the innate immune system.^109^ The results from *in vitro* studies have demonstrated innate immunity upon *in vitro* application of gRNA, leading to up-regulation of the interferon response and ultimately cell death.^110^^,^^111^ This can be mitigated using synthetic gRNA or chemical modification of *in vitro* transcribed gRNAs^110^^,^^111^ or targeting of the molecular interferon response. Further avenues await exploration to identify optimal methods to avoid the innate immune response to gRNA, which is delivered in mRNA and DNA forms.

Delivery of SaCas9 by AAV has been shown to lead to the development of anti-SaCas9 antibodies following systemic delivery in adult mice and after intravitreal delivery in non-human primates.^112^ On the other hand, systemic injection of neonatal mice with AAV-SaCas9 did not result in antibody development,^113^ as their humoral immune response has not yet developed, which highlights the effect of the age of the animal on immune response development.^113^^,^^114^ In addition, the complement pathway has been shown to contribute to the immunogenicity of the AAV vector in human blood,^115^ which may be mitigated by blocking the complement pathway and decreasing the immunogenicity of AAV-based therapeutics. Publicly reported clinical cases of complement-mediated thrombotic microangiopathy associated with systemic AAV gene transfer have been provided in a comprehensive review.^116^ These cases have been observed across a range of patient populations and involve various AAV capsid types. This article represents a significant advance in understanding the safety profile of AAV gene therapy, promoting greater transparency and data sharing to facilitate the development of risk mitigation strategies for this serious adverse event.^116^ Recent findings reported the death of a 27-year-old Duchenne muscular dystrophy patient as a result of an innate immune reaction that caused acute respiratory distress syndrome following the administration of 1 × 10^14^ recombinant AAV vector genomes per kilogram of body weight.^117^ In this study, the patient was treated with recombinant AAV serotype 9 containing dSaCas9 fused to the VP64 transgene, which is designed to up-regulate cortical dystrophin, as a custom CRISPR-transactivator therapy.^117^ As both innate and adaptive immunity can occur following the administration of CRISPR‒Cas, monitoring such responses during the clinical development of CRISPR therapeutics is necessary.

In a study by Chew et al, Cas9-expressing muscles presented increased numbers of CD45^+^ leukocytes, specifically myeloid cells, including monocytes, macrophages, and dendritic cells. This observation was accompanied by varying titers of Cas9-specific antibodies in individual mice following Cas9 administration. This Cas9-mediated immune response occurred regardless of the delivery method.^112^ Cas9 epitope mapping revealed the presence of three linear epitopes that play essential roles in Cas9 function, including gRNA recognizing residues, the REC-1 domain, which contributes to the Cas9-gRNA interaction, and the PAM binding loop. The concordance of the adaptive immune response in the human body against Cas9 orthologs was further demonstrated by another group, where human serum was probed for the presence of anti-Cas9 antibodies.^118^ Anti-SaCas9 and anti-SpCas9 antibodies have been detected in human serum samples in two separate studies.^118^^,^^119^ Although these studies investigate different sample sizes and use different methods of detection, they both report pre-existing antibodies to the two most popular Cas9 orthologs, which could ultimately affect their *in vivo* application.

The presence of antigen-reactive T cells is another indication of a pre-existing immune response to Cas9. The results from a study identifying pre-existing adaptive immunity to Cas9 proteins in humans confirmed a high frequency of cytokine-positive antigen-reactive T cells against SaCas9 (78% donors) and SpCas9 (67% donors). All donors positive for antibody reactivity were also positive for cellular activity.^118^ The results from these studies suggest that screening for anti-Cas9 antibodies and Cas9 TCR-expressing T cells would be advisable prior to any *in vivo* gene editing. As there is an increased risk of *Staphylococcus* infections in hospitals,^120^^,^^121^ dormant anti-Cas9 cytotoxic T lymphocytes may become activated in patients receiving treatment, leading to ineffective treatment. An option to avoid a T-cell response would be immune suppression using anti-inflammatory or immunomodulatory drugs, which ameliorate the immune response against CRISPR/Cas9; however, this would not be recommended for long-term treatment.

The up-regulation of CD137 in both CD4^+^ T-cell and CD8^+^ T-cell subpopulations following SpCas9 administration demonstrates the activation of T effector (Teff) cells. Interestingly, SpCas9 has been shown to induce an effector-memory T-cell response accompanied by an increase in regulatory T (Treg) cells in humans.^122^ *In vitro* studies also demonstrate that endogenous SpCas9-reactive Treg cells have the potential to reduce the activation, expansion, and function of SpCas9-reactive Teff cells. Therefore, screening for alterations in the Treg/Teff cell ratio and strong CD8^+^ T-cell responses could be used to assess pre-existing immunity to Cas9 and may be beneficial prior to *in vivo* delivery of CRISPR/Cas9 components. Considering the inhibitory effect of Treg cells on T-cell activation, Treg cell-stimulating peptides may help control the overall immune response for *in vivo* delivery of gene therapy. When Treg peptides are administered, the number of Cas9-specific Treg cells in the host increases. Similarly, *ex vivo* expanded Cas9-specific Treg cells from patients could be reinjected before *in vivo* gene editing. However, more studies have explored the safety and effectiveness of these options.

To minimize the chance of an adverse immune response following the administration of *in vivo* CRISPR/Cas9 gene therapy, certain strategies can be utilized to evade pre-existing immunity to Cas9. Among these structural modifications to the Cas9 nuclease, masking immunogenic epitopes is an interesting approach. Ferdosi et al identified two immunodominant SpCas9 T-cell epitopes for HLA-A∗0.2:01 via a combined *in silico* and *in vitro* system. In this study, the authors utilized an enhanced prediction algorithm that incorporates T-cell receptor contact residue hydrophobicity and HLA binding and evaluated them by T-cell assays using healthy donor peripheral blood mononuclear cells. In this approach, Ferdosi et al eliminate the immunodominant epitopes and demonstrate that the resulting mutated Cas9 has a 20–30-fold reduction in T-cell reactivity while maintaining its nuclease activity.^123^

The Epstein–Barr virus-encoded nuclear antigen (EBNA1) is expressed in latent Epstein–Barr virus-infected B lymphocytes that persist for life in healthy viral carriers.^124^ It has been demonstrated that generating glycine–alanine (Gly–Ala) repeats upstream of the 416–424 epitope of EBNA1 generates a cis-acting inhibitory signal that interferes with antigen processing and MHC class I-restricted presentation.^125^ Based on this, altering the antigen presentation of Cas9 epitopes might restrict the proteasomal degradation of Cas9 and antigen presentation to cytotoxic T lymphocytes via HLA class I in a similar manner.^126^ The unanswered question is whether this approach has any inhibitory effects on the functionality of the Cas9 nuclease.

Other approaches for increasing the efficiency of CRISPR/Cas gene editing and reducing toxicity concerns associated with this technique include utilizing immune suppression, using Cas9 orthologs from non-pathogenic bacteria, targeting immune-privileged organs, and inducing immune tolerance. As the delivery vector plays an important role in the activation of immunity, it is essential to mitigate the immune response to both Cas9 and the delivery vehicle to ensure efficacious gene editing. As discussed earlier, the use of non-viral vectors may mitigate the risk of immunogenicity associated with the *in vivo* application of CRISPR/Cas9, which is further influenced by the clinical indication and route of administration.

## Concluding remarks

Non-clinical studies are conducted to provide scientific insight into the efficacy and safety of novel therapeutics and are critical for assessing risk: a beneficial relationship with clinical relevance. “*The evaluation of investigational CGT products necessitates a careful risk-benefit analysis performed in the context of the clinical indication under study*” (FDA-2012-D-1038).^13^ Considering the non-homogeneous characteristics of CGTs, a “*case-by-case risk-based approach should be taken when designing non-clinical testing programs*” (EMA/CAT/CPWP/686637/2011).^127^ This approach addresses various factors related to the quality, biological activity, and clinical application of CGTs. These factors include the specific properties of the CGT, route of administration, target and non-target tissues/organs, and disease status of the patient population. Once risks have been identified, efficacy, *in vivo* safety, biodistribution, and persistence studies need to be performed in a suitable non-clinical species prior to the commencement of clinical investigations. Where appropriate, this may also constitute a paper-based exercise whereby published scientific literature can be used to address a specific safety concern. By determining the risks for an investigational CGT, the extent of any non-clinical package can be determined. The non-clinical risk profile of a CGT should be revisited as product development proceeds. The overall objectives that the non-clinical program needs to address are the establishment of biological plausibility, identification of/support of clinical dose levels, feasibility and safety of the proposed route of administration, and general safety of the product. These studies aim to generate clinically translatable data to support the safe use of the product in the intended clinical indications. To facilitate a smoother bench-to-bedside transition of a CRISPR/Cas-modified CGT, it is essential to have a defined clinical indication, an intended clinical population, a clinical route of administration, and specifics on the drug product components as early as possible in drug development.

In this article, we have reviewed recent advances in the use of CRISPR/Cas technology in gene/genome editing. Despite the rapid development of this technique, there remain critical safety concerns that need to be addressed and mitigated when CGT is generated via these methods. Here, we have discussed CRISPR/Cas clinical trial data, highlighted the risks associated with CRISPR/Cas technology, and provided possible mitigations to capture these risks in non-clinical studies. Because *in vivo* CRISPR/Cas gene editing is associated with greater risks compared with the *in vitro* method, a more extensive risk assessment should be performed to capture DNA damage toxicity, tumorigenicity, off-target editing, and immunogenicity.

As a final word, and for a more facilitated path to the clinic, we recommend that scientists have a reasonable and sound approach to the appropriate risks and follow appropriate guidelines such as the “*Guideline on non-clinical studies required before first clinical use of gene therapy medicinal products*” (FDA-2012-D-1038, EMEA/CHMP/GTWP/125459/2006)^13^^,^^14^ and, where applicable, ICH E11(R1) “*Guideline on clinical investigation of medicinal products in the pediatric population*” (EMA/CPMP/ICH/2711/1999)^17^ as early as possible in the development of the investigational CGT.

## CRediT authorship contribution statement

**Parto Toofan:** Writing – review & editing, Writing – original draft, Project administration, Investigation, Data curation, Conceptualization. **Mark Singh:** Writing – review & editing. **Andrew Brooks:** Writing – review & editing. **Keith McLuckie:** Writing – review & editing, Supervision, Conceptualization.

## Conflict of interests

The authors declare that they have no declarations of interests and are full-time employees of The Cell and Gene Therapy Catapult.
