# Supplementary material for: Non-clinical safety considerations on genome editing using the CRISPR/Cas system
Source: Genes Dis. 2025 Jul 28;13(2):101785. doi: 10.1016/j.gendis.2025.101785 (PMC12682025; doi:10.1016/j.gendis.2025.101785)
Supplement: Multimedia component 1 [file mmc1.docx]

**Table S1. List of clinical trials utilizing CRISPR/Cas9 to generate/modify cell and gene therapy products.** Data extracted from GlobalData and ClinicalTrials.gov and combined on 13 March 2025. Acute Lymphocytic Leukemia (ALL), Acute Myelocytic Leukemia (AML), Adeno Associated Virus (AAV), Anaplastic Large Cell Lymphoma (ALCL), Angioimmunoblastic T-Cell Lymphoma (AITL), Bone Marrow (BM), C1 Esterase Inhibitor (C1-INH), Castration-Resistant Prostate Cancer (CRPC), C-C chemokine receptor type 5 (CCR5), Centrosomal Protein of 290 kDa (CEP290), Cervical Intraepithelial Neoplasia (CIN), Chimeric antigen receptor T-cell (CAR T-cell), Chronic Lymphocytic Leukemia (CLL), Clustered Regularly Interspaced Short Palindromic Repeats and CRISPR-Associated Protein 9 (CRISPR/cas9), Coronary Artery Disease (CAD), Cytokine Inducible SH2 Containing Protein (CISH), Epidermal growth factor receptor (EGFR), Fanconi Anemia Complementation group C (FANCC), Fetal hemoglobin (HbF), Gastrointestinal (GI), Hemoglobin Subunit Beta (HBB), Hemopoietic stem and progenitor cells (HSPCs), Hemopoietic stem cells (HSCs), Hereditary Angioedema (HAE), Heterozygous familial hypercholesterolemia (heFH), HIV (human immunodeficiency virus), Human Papillomavirus (HPV), Intravenous (IV), Kallikrein B1 (KLKB1), Knock Out (KO), Leber Congenital Amaurosis (LCA), Lentiviral (LV), Long Terminal Repeat (LTR), Multiple Myeloma (MM), Muscle Invasive Bladder Cancer (MIBC),Non-Hodgkin Lymphoma (NHL), Non-Small Cell Lung Cancer (NSCLC), Pancreatic Endoderm Cells (PECs), Peripheral T-Cell Lymphomas (PTCL), Programmed cell Death protein 1 (PD-1), Route of Administration (ROA), Simplexvirus (HSV), single-chain variable fragment (scFv), Subcutaneous (SQ), T-Cell Receptor (TCR), Transforming Growth Factor Β Receptor (TGFβR), Transthyretin (TTR), Tumor Infiltrating Lymphocytes (TIL), Vascular Endothelial Growth Factor A (VEFGA), Wilms Tumor 1 (WT1).

| **Trial ID** | **Trial Phase** | **Intervention/ Treatment** | **Target** | **Mode** | **Therapy Area** | **Clinical Indication** | **ROA** | **Trial Status** | **Delivery method** |
| --- | --- | --- | --- | --- | --- | --- | --- | --- | --- |
| NCT06262399 | IV | LNP-based CRISPR/Cas9 delivery system which comprises of guide RNA specific to the disease-causing gene and messenger RNA | KLKB1 gene | *In vivo* | Immunology | HAE, C1-INH Deficiency | IV | Ongoing, recruiting by invitation | Undefined |
| NCT05356195 | III | autologous CRISPR-Cas9 modified CD34+ human hematopoietic stem and progenitor cells (hHSPCs | BCL11A gene | *Ex vivo* | Hematological Disorders | Β Thalassaemia, Anemia, Sickle Cell Disease,Thalassemia, Hemoglobinopathies, Unspecified Genetic Disorders, Unspecified Hematological Disorders | IV infusion | Ongoing, recruiting by invitation | Undefined |
| NCT05951205 | III | autologous CD34+ hHSPCs modified with CRISPR-Cas9 at the erythroid lineage-specific enhancer of the target gene | BCL11A gene | *Ex vivo* | Hematological Disorders | Sickle Cell Disease | IV infusion | Planned | Undefined |
| NCT05329649 | III | autologous CD34+ hHSPCs modified with CRISPR-Cas9 at the erythroid lineage-specific enhancer of the target gene | BCL11A gene | *Ex vivo* | Hematological Disorders | Sickle Cell Disease, Hemoglobinopathies | IV infusion | Ongoing, recruiting | Undefined |
| NCT06465537 | II | modified third-generation integrated defective lentivirus, that delivers gRNA and mRNA encoding Cas9 RNP complex RNP that silences the mutated target gene | MYOC gene | *In vivo* | Ophthalmology | Open-Angle Glaucoma | Intracameral | Ongoing, recruiting | Modified third generation integrated defective lentivirus |
| NCT05120830 | I/II | LNP-based CRISPR/Cas9 delivery system which comprises of guide RNA specific to the disease-causing gene and messenger RNA | KLKB1 gene | *In vivo* | Immunology | HAE, C1-INH Deficiency | IV | Ongoing, not recruiting | Undefined |
| NCT06379789 | I/II | LNP-based CRISPR/Cas9 delivered using AAV | Factor IX Gene | *In vivo* | Hematological Disorders | Hemophilia B (Factor IX Deficiency) | IV | Ongoing | AAV |
| NCT03164135 | I | CRISPR/Cas9 modified CD34+ hematopoietic stem/progenitor cells | CCR5 | *Ex vivo* | Infectious Disease; Oncology | HIV Infections AIDS, Hematological Tumor, ALL, Acute Lymphoblastic Leukemia | Parenteral | Completed | Undefined |
| NCT06506461 | I | CRISPR/Cas9 (RNP) edited CD34+ hematopoietic stem and progenitor cells | - | *Ex vivo* | Hematological Disorders | Sickle Cell Disease | IV infusion | Ongoing, recruiting | Undefined |
| NCT04502446 | I | Allogeneic CRISPR-Cas9-engineered T Cells | CD70 Antigen (CD27 Ligand or Tumor Necrosis Factor Ligand Superfamily Member 7 or CD70) | *Ex vivo* | Oncology | Diffuse Large B-Cell Lymphoma, T-Cell Lymphomas, Unspecified B-Cell Lymphomas | IV | Terminated | Undefined |
| NCT06031727 | I | CRISPR/cas13-medIated RNA targeting therapy | Vascular Endothelial Growth Factor A (HG202) | *In vivo* | Ophthalmology | Age Related Macular Degeneration, Choroidal Neovascularization, Wet (Neovascular / Exudative) Macular Degeneration | Parenteral | Ongoing, recruiting | Undefined |
| NCT05566223 | I/II | CRISPR/Cas9 modified TILs | Gene Encoding CISH | *Ex vivo* | Oncology | Lung Adenocarcinoma, NSCLC | IV | Withdrawn | Undefined |
| NCT06492304 | I/II | Allogeneic CRISPR-Cas9 gene edited T cells | CD70 Antigen | *Ex vivo* | Oncology | Follicular Lymphoma, Sezary Syndrome, Hematological Tumor, AML, Myelodysplastic Syndrome (Preleukemia), Diffuse Large B-Cell Lymphoma, T-Cell Lymphomas, ALCL, AITL/Immunoblastic Lymphadenopathy, Mantle Cell Lymphoma, Marginal Zone B-Cell Lymphoma, Mycosis Fungoides, PTCL, Unspecified B-Cell Lymphomas | IV | Ongoing, recruiting | Undefined |
| NCT05643742 | I/II | Allogeneic CRISPR-Cas9-Engineered T Cells | CD19 | *Ex vivo* | Oncology | Follicular Lymphoma, B-Cell NHL, B-Cell, Lymphoma, NHL, CLL, Diffuse Large B-Cell Lymphoma, Mantle Cell Lymphoma, Marginal Zone B-Cell Lymphoma | IV | Ongoing, recruiting | Undefined |
| NCT05477563 | III | CRISPR Cas9 modified CD34+ human hematopoietic stem and progenitor cells | BCL11A | *Ex vivo* | Genetic Disorders; Hematological Disorders | βThalassaemia, Anemia, Sickle Cell Disease, Thalassemia, Hemoglobinopathies, Unspecified Genetic Disorders, Unspecified Hematological Disorders | IV | Ongoing, recruiting | Undefined |
| NCT04208529 | III | Autologous CRISPR-Cas9 Modified CD34+ human hematopoietic stem and progenitor cells | BCL11A | *Ex vivo* | Genetic Disorders; Hematological Disorders | β Thalassaemia, Anemia, Sickle Cell Disease, Thalassemia, Hemoglobinopathies, Unspecified Genetic Disorders, Unspecified Hematological Disorders | IV | Ongoing, recruiting by invitation |  |
| NCT06128629 | III | CRISPR/Cas9 and gRNA packed in LNP | TTR gene | *Ex vivo* | Metabolic Disorders | Amyloidosis, Familial Amyloid Cardiomyopathy, Amyloid Cardiomyopathy | IV/Parenteral | Ongoing, recruiting |  |
| NCT03745287 | II/III | Autologous CRISPR-Cas9 Modified CD34+ Human Hematopoietic Stem and Progenitor Cells | BCL11A gene | *Ex vivo* | Hematological Disorders | β Thalassaemia, Anemia, Sickle Cell Disease, Thalassemia, Hemoglobinopathies, Unspecified Genetic Disorders, Unspecified Hematological Disorders | IV | Ongoing, recruiting by invitation |  |
| NCT03655678 | II/III | Autologous CRISPR-Cas9 Modified CD34+ Human Hematopoietic Stem and Progenitor Cells | BCL11A gene | *Ex vivo* | Genetic Disorders; Hematological Disorders | β Thalassaemia, Sickle Cell Disease, Thalassemia, Hemoglobinopathies, Sickle Cell Disease with Vaso-Occlusive Crisis, Unspecified Genetic Disorders, Unspecified Hematological Disorders | IV | Ongoing, not recruiting |  |
| NCT04443907 | I/II | LNP-based CRISPR/Cas9 gene edited bone marrow-derived CD34 positive hematopoietic stem cells | BCL11A gene | *Ex vivo* | Hematological Disorders | Sickle Cell Disease | IV | Completed |  |
| NCT04819841 | I/II | CRISPR-Cas9 genome edited, autologous CD34+ hematopoietic stem cells | HbS to HbA gene correction | *Ex vivo* | Hematological Disorders | Sickle Cell Disease | IV | Ongoing, recruiting |  |
| NCT06155500 | I/II | Autologous HSPCs | BCL11A | *Ex vivo* | Hematological Disorders | Sickle Cell Disease | IV infusion | Ongoing, recruiting |  |
| NCT05144386 | I/II | CRISPR/Cas9 and gRNAs directed using AAV9 vector | HIV-1 LTR U3 | *Ex vivo* | Infectious Disease | HIV Infections (AIDS) | IV | Completed | AAV |
| NCT04774536 | I/II | Biologic; Gene-Modified Cell Therapy; Small Molecule |  |  | Hematological Disorders | Sickle Cell Disease |  | ongoing |  |
| NCT04557436 | I | Biologic; Gene-Modified Cell Therapy |  |  | Oncology | B-Cell Acute Lymphocytic Leukemia |  | Completed |  |
| GDCT0267661 | I | AAV9 delivered CRISPR/Cas9 guide RNAs | HIV-1 LTR U3 upstream promoter region | *Ex vivo* | Infectious Disease | HIV Infections (AIDS) | IV | Withdrawn | AAV9 |
| NCT05143307 | I | Biologic; Gene Therapy |  |  | Infectious Disease | HIV Infections (AIDS) |  | Ongoing, recruiting by invitation |  |
| NCT04601051 | I | Biologic; Gene Therapy |  |  | Cardiovascular; Metabolic Disorders | Amyloidosis, Cardiomyopathy, Familial Amyloid Cardiomyopathy, Familial Amyloid Neuropathies, Polyneuropathies |  | Ongoing, not recruiting |  |
| NCT04438083 | I | Biologic; Gene-Modified Cell Therapy; Small Molecule |  |  | Oncology | Renal Cell Carcinoma, Hematological Tumor, Solid Tumor, Metastatic Renal Cell Carcinoma |  | Terminated |  |
| GDC20006929 | I/II | CRISPR/Cas9 and gRNA delivered by AAV9 vector | FANCC | *Ex vivo* | Hematological Disorders | Fanconi Anemia | Unspecified | Withdrawn | AAV |
| GDC30015205 | I/II | CRISPR/Cas9 and gRNA RNP delivered by AAV9 vector |  | *Ex vivo* | Hematological Disorders | Unspecified Hematological Disorders | Unspecified | Withdrawn | AAV |
| NCT04560790 | I/II | CRISPR/CAS9 components administered in mRNA format for the treatment of viral keratitis caused by herpes simplex virus | Unspecified | *In vivo* | Ophthalmology Infectious disease | HSV Infections, Herpetic Keratitis, Viral Keratitis | Intracorneal | Completed | mRNA |
| NCT03166878 | I/II | Allogeneic CAR-T Cells | CD19 | *Ex vivo* | Oncology | B-Cell Leukemia, Unspecified B-Cell Lymphomas | IV | Terminated | Undefined |
| NCT03398967 | I/II | Autologous CAR-T containing anti CD19 and anti CD20 scFv transduced with retroviral vector. | CD19, CD20 | *Ex vivo* | Oncology | Lymphoma, Leukemia, B-Cell Leukemia, Unspecified B-Cell Lymphomas | Devic's Syndrome: IV, BCL: Parenteral | Ongoing, recruiting | Undefined, likely electroporation |
| NCT04426669 | I/II | Autologous TILs | CISH | *Ex vivo* | Oncology | Gallbladder Cancer, Esophageal Cancer, GI Tract Cancer, Epithelial Tumor, Colorectal Cancer, Pancreatic Cancer, Gastric Cancer, Colon Cancer, GI Tumor | SQ | Ongoing, not recruiting | Transient Cas9 |
| NCT05066165 | I/II | Autologous immune cells | WT1 receptor on cancer cells | *Ex vivo* | Oncology | AML | IV | Terminated | Undefined |
| NCT04976218 | I | Autologous CAR-EGFR-TGFβR-KO T cells | EGFR, TGFβR | *Ex vivo* | Oncology | Solid tumor | Parenteral as infusion | Ongoing, recruiting | Undefined, likely electroporation |
| NCT05210530 | I | Allogeneic PECs on a delivery device for β cell replacement therapy | Unspecified | *Ex vivo* | Immunology and Metabolic Disorders | Type 1 Diabetes (Juvenile Diabetes), Autoimmune Disorders | Unavailable | Completed | Undefined, likely electroporation |
| NCT04637763 | I | Allogeneic CD19 CAR-T | PD-1 KO | *Ex vivo* | Oncology | Follicular Lymphoma, B-Cell, NHL, Diffuse Large B-Cell Lymphoma, Mantle Cell Lymphoma, Marginal Zone B-Cell Lymphoma, Primary Mediastinal B-Cell Lymphoma | Parenteral | Ongoing, recruiting | Electroporation mRNA |
| NCT02793856 | I | Allogeneic CD19 CAR-T | PD-1 KO | *Ex vivo* | Oncology | NSCLC | Parenteral | Completed | Undefined, likely electroporation |
| NCT03044743 | I/II | Autologous T cells | PD-1 KO | *Ex vivo* | Oncology, Infectious Disease | Nasopharyngeal Cancer, Hodgkin Lymphoma (B-Cell Hodgkin Lymphoma), Gastric Cancer, Diffuse Large B-Cell Lymphoma, T-Cell Lymphomas, Epstein–Barr Virus (HHV-4) Infections | Parenteral | Completed | Electroporation mRNA |
| NCT03081715 | I | Autologous T cells | PD-1 KO | *Ex vivo* | Oncology | Esophageal Cancer | Parenteral | Completed | Undefined |
| NCT04244656 | I | Allogeneic CAR-T | BCMA | *Ex vivo* | Oncology | MM (Kahler Disease), Refractory MM, Relapsed MM | IV | Completed | Plasmid Delivery |
| NCT04035434 | I | Allogeneic CD19 CAR-T | CD19 | *Ex vivo* | Oncology | B-Cell ALL, Follicular Lymphoma, B-Cell NHL, B-Cell Chronic Lymphocytic Leukemia, NHL, ALL, , CLL, Diffuse Large B-Cell Lymphoma | IV | Terminated | Undefined |
| NCT03690011 | I | Autologous T cells expressing CAR conjugated with CD28 protein | CD7 KO | *Ex vivo* | Oncology | T-Cell Acute Lymphocytic Leukemia (T-Cell Acute Lymphoblastic Leukaemia), T-Cell Leukemia, Sezary Syndrome, NHL, ALL, T-Cell Lymphomas, ALCL, AITL/Immunoblastic Lymphadenopathy, Mycosis Fungoides, Natural Killer Cell Lymphomas, PTCL | Parenteral | Ongoing, recruiting | Undefined |
| NCT04037566 | I | Autologous CD19 CAR-T Cells, | Disruption of HPK1 | *Ex vivo* | Oncology | Burkitt Lymphoma, Follicular Lymphoma, Waldenstrom Macroglobulinemia (Lymphoplasmacytic Lymphoma), ALL, CLL, Diffuse Large B-Cell Lymphoma, Hairy Cell Leukemia, Mantle Cell Lymphoma, Marginal Zone B-Cell Lymphoma, Unspecified B-Cell Lymphomas | IV | Completed | Undefined |
| NCT04925206 | I | Autologous CD34+ HSPCs | BCL11A | *Ex vivo* | Hematological Disorders | β Thalassaemia | IV | Ongoing, not recruiting | Undefined |
| NCT03872479 | I/II | CRISPR/Cas9 delivered using AAV5 | CEP290 gene in retinal cells | *In vivo* | Ophthalmology | LCA, Retinal Degeneration, Unspecified Ophthalmological Disorders, Vision Disorders | Intraocular | Ongoing, not recruiting | Subretinal injection of AAV5 encoding sgRNA and SaCas9 |
| NCT03057912 | I | TALEN and CRISPR/Cas9 components delivered as plasmids in gel | Inactivating E6 and E7 proteins of HPV | *In vivo* | Oncology | HPV Associated Cancer, CIN, Malignant Neoplasms | Unspecified | Withdrawn | - |
| NCT03019666 | I/II | Allogeneic NAM-NK cells | Unspecified | *Ex vivo* | Oncology | Follicular Lymphoma, Waldenstrom Macroglobulinemia (Lymphoplasmacytic Lymphoma), B-Cell NHL, Lymphoma, MM (Kahler Disease), NHL, Diffuse Large B-Cell Lymphoma, Mantle Cell Lymphoma, Primary Mediastinal B-Cell Lymphoma, Refractory MM, Relapsed MM | IV | Completed | Undefined, likely electroporation |
| NCT04614636 | I | CRISPR/Cas9 edited iPSC derived NK cells | CD38 KO | *Ex vivo* | Oncology | Blood Cancer, MM (Kahler Disease, AML), Refractory Acute Myeloid Leukemia, Refractory MM, Relapsed Acute Myeloid Leukemia, Relapsed MM | IV | Terminated | Undefined, likely electroporation |
| NCT04026100 | I/II | Allogeneic CD19 CAR-T | CD19 | *Ex vivo* | Oncology | Diffuse Large B-Cell Lymphoma | IV | Withdrawn | Undefined, likely electroporation |
| NCT03399448 | I | Autologous T Cells Engineered to express NY-ESO-1 TCR | TCR and PD-1 KO (NYCE T Cells) | *Ex vivo* | Oncology | Melanoma, MM (Kahler Disease), Sarcomas, Myxoid Liposarcoma, Refractory MM, Relapsed MM, Round Cell Liposarcoma, Synovial Sarcoma | Unspecified | Terminated | Undefined, likely electroporation |
| NCT02863913 | I | T cells | PD-1 KO | *Ex vivo* | Oncology | Bladder Cancer, MIBC | Parenteral | Withdrawn | Undefined, likely electroporation |
| NCT02867332 | I | T cells | PD-1 KO | *Ex vivo* | Oncology | Metastatic Renal Cell Carcinoma | Parenteral | Withdrawn | Delivery Vector |
| NCT02867345 | I | T cells | PD-1 KO | *Ex vivo* | Oncology | CRPC | Parenteral | Withdrawn | Electroporation mRNA |
| NCT03538613 | I/II | TILs | CISH KO | *Ex vivo* | Oncology | Gallbladder Cancer, Esophageal Cancer, GI Tract Cancer, Epithelial Tumor, Colorectal Cancer, Pancreatic Cancer, Gastric Cancer, Colon Cancer, Gastrointestinal Tumor | IV | Ongoing, not recruiting | Electroporation mRNA |
| NCT03728322 | 0 | Autologous CD34+ HSPCs | HBB | *Ex vivo* | Hematological Disorders | β Thalassaemia, Thalassemia | IV | Withdrawn | Electroporation mRNA |
| NCT03747965 | I | CAR-T | PD-1 KO | *Ex vivo* | Oncology | Ovarian Cancer, Bile Duct Cancer (Cholangiocarcinoma), Colorectal Cancer, Pancreatic Cancer, Solid Tumor | Parenteral | Completed | Undefined |
| NCT04089891 | I/II | TILs | CISH KO | *Ex vivo* | Oncology | Gallbladder Cancer, Esophageal Cancer,GI Tract Cancer, Epithelial Tumor, Colorectal Cancer, Pancreatic Cancer, Gastric Cancer, Colon Cancer, GI Tumor | SQ | Ongoing, not recruiting | Undefined |
| NCT04853576 | I/II | Autologous CRISPR/Cas12a gene edited CD34+ HSCs | HbF | *Ex vivo* | Hematological Disorders | Sickle Cell Disease, Hemoglobinopathies | IV infusion | Completed | AAV, RNP |
| NCT05444894 | I/II | Autologous CRISPR/Cas12a gene edited CD34+ HSCs | HbF | *Ex vivo* | Hematological Disorders | β Thalassaemia, Thalassemia, Hemoglobinopathies | IV | Terminated | AAV, RNP |
| NCT05398029 | I | Base editing using CRISPR/Cas9 | Base editing of PCSK9 | *In vivo* | Metabolic Disorders, Cardiovascular | Cardiovascular Disease, Hypercholesterolemia, Atherosclerosis, heFH | IV | Ongoing, not recruiting | Base editing, LNP mediated delivery |
| NCT06164730 | I | Base editing using CRISPR/Cas9 | Base editing of PCSK9 | *In vivo* | Metabolic Disorders, Cardiovascular | CAD (ischemic heart disease), heFH | IV | Ongoing, recruiting | Base editing, GalNAc-LNP delivery |
| NCT06737146 | I | CRISPR/Cas9 edited B7H3-specific allogeneic CAR-T cells | CD276 | *Ex vivo* | Oncology | High-Grade Glioma | Intracerebral; Intrapleural; Intrathecal | Planned | Undefined |
| NCT06742593 | I | CRISPR/Cas9 edited B7H3-specific allogeneic CAR-T cells. | CD276 | *Ex vivo* | Oncology | Breast Cancer, Lung Cancer, Renal Cell Carcinoma, Colorectal Cancer, Melanoma, Solid Tumor, High-Grade Glioma | Intracerebral; Intrapleural; Intrathecal | Planned | Undefined |
| NCT06672237 | III | CRISPR/Cas9 guide RNAs directed to transthyretin (TTR) gene, packed in a lipid nanoparticle | TTR gene | *In vivo* | Metabolic Disorders, Central Nervous System, Genetic Disorders | Amyloidosis, Familial Amyloid Neuropathies, Inherited Neurodegenerative Diseases, Neurodegenerative Diseases, Neuromuscular Disorders, Polyneuropathies, Unspecified Genetic Disorders, Unspecified Neurologic Disorders | IV | Ongoing, recruiting | LNP mediated delivery |
| NCT06634420 | III | CRISPR/Cas9 guide RNA directed to KLKB1 gene, packed in a lipid nanoparticle | KLKB1 gene | *In vivo* | Immunology | HAE (C1-INH Deficiency) | IV | Ongoing, recruiting | LNP mediated delivery |
